# Impact of Mitral Regurgitation Recurrence on Mitral Valve Repair for Secondary Ischemic Mitral Regurgitation

**DOI:** 10.3390/jcdd10030124

**Published:** 2023-03-15

**Authors:** Antonio Salsano, Antonio Nenna, Nicolas Molinari, Sanjeet Singh Avtaar Singh, Cristiano Spadaccio, Francesco Santini, Massimo Chello, Antonio Fiore, Francesco Nappi

**Affiliations:** 1 DISC Department, University of Genoa, 16132 Genova, Italy; 2 Department of Cardiovascular Surgery, Università Campus Bio-Medico di Roma, 00128 Rome, Italy; 3 IDESP, INSERM, PreMEdical INRIA, University of Montpellier, CHU Montpellier, 34295 Montpellier, France; 4 Cardiothoracic Surgery, Royal Infirmary of Edinburgh, Edinburgh EH16 4SA, UK; 5 Department of Cardiovascular Surgery, Mayo Clinic, Rochester, MN 55905, USA; 6 Department of Cardiac Surgery, Hôpitaux Universitaires Henri Mondor APHP, 94000 Creteil, France; 7 Advanced Surgical Technologies, Sapienza University of Rome, 00128 Roma, Italy; 8 Cardiac Surgery, Centre Cardiologique du Nord de Saint-Denis, 93200 Paris, France

**Keywords:** secondary mitral regurgitation, papillary muscle, left ventricular remodeling, ischemic heart disease, mitral valve repair

## Abstract

Objectives. The current guidelines still do not include specific recommendations on the use of subvalvular repair (SV-r) for treatment of ischemic mitral regurgitation (IMR). Therefore, the objective of our study was to evaluate the clinical impact of mitral regurgitation (MR) recurrence and ventricular remodeling on long-term outcomes after SV-r combined with restrictive annuloplasty (RA-r). Methods. We performed a subanalysis of the papillary muscle approximation trial, studying 96 patients with severe IMR and coronary artery disease undergoing restrictive annuloplasty alongside subvalvular repair (SV-r + RA-r group) or restrictive annuloplasty alone (RA-r group). We analyzed treatment failure differences, the influence of residual MR, left ventricular remodeling, and clinical outcomes. The primary endpoint was treatment failure (composite of death; reoperation; or recurrence of moderate, moderate-to-severe, or severe MR) within 5 years of follow-up after the procedure. Results. A total of 45 patients showed failure of the treatment within 5 years, of which 16 patients underwent SV-r + RA-r (35.6%) and 29 underwent RA-r (64.4%, *p* = 0.006). Patients with significant residual MR presented with a higher rate of all-cause mortality at 5 years compared with trivial MR (HR 9.09, 95% CI 2.08–33.33, *p* = 0.003). MR progression occurred earlier in the RA-r group, as 20 patients in the RA-r group vs. 6 in SV-r + RA-r group had a significant MR 2 years after surgery (*p* = 0.002). Conclusions. RA-r remains a surgical mitral repair technique with an increased risk of failure and mortality at 5 years compared with SV-r. The rates of recurrent MR are higher, and recurrence occurs earlier, with RA-r alone compared to SV-r. The addition of the subvalvular repair increases the durability of the repair, thus extending all of the benefits of preventing MR recurrence.

## 1. Introduction

Ischemic mitral regurgitation (IMR) is a clinicopathological perturbance associated with significant morbidity and mortality [1,2,3,4,5]. Patients who develop severe IMR experience a serious prognosis, with rates of death ranging from 15 to 40% at 1 year [6,7,8]. IMR affects approximately 10% of patients experiencing a myocardial infarction (MI) [9,10]. The latter determines substantial changes in left ventricular (LV) geometric configuration, resulting in distortion of the normal spatial relationships of the constituents of the mitral valve (MV) apparatus, which results in an incomplete mitral leaflet coaptation and secondary mitral regurgitation (SMR) [1,2,3,4,5]. The set of these alterations exacerbates maladaptive LV remodeling, leading to the development of heart failure (HF), decreased quality of life (QOL), and reduced survival [1,2,3,4,5,11,12].

The current guidelines still do not include specific recommendations on the use of subvalvular repair (SV-r) combined with restrictive annuloplasty repair (RA-r) in the treatment of IMR. This procedure has not been widely endorsed by the surgical community, and the most common surgical indications for SMR remain chordal-sparing mitral valve replacement (MVR) and restrictive annuloplasty [13,14]. The use of SV-r associated with RA-r compared to RA-r alone has been shown to be superior in achieving ventricular remodeling with lower mitral regurgitation (MR) recurrence and improved short-term survival in prospective randomized [4,5,15,16] and non-randomized studies [17]. However, despite the literature supporting its use, a certain degree of resistance to the wider use of subvalvular repair is encountered in the surgical community; therefore, it requires validation from randomized clinical trials (RCTs) and more robust data on long-term outcomes and failure modes.

In the papillary muscle approximation (PMA) randomized trial, 96 patients with severe chronic ischemic mitral regurgitation underwent complete surgical myocardial revascularization, associated with either isolated RA-r or SV-r plus RA-r, and were followed up on for 5 years. Left ventricular end-diastolic diameter improved at the 5-year follow-up (5.8 ± 4.1 mm and −0.2 ± 2.3 mm, respectively; *p* < 0.001), maintaining the benefit achieved immediately postoperatively with freedom from major adverse cardiac and cerebrovascular events (*p* = 0.004) [3].

The objective of our study was to evaluate the clinical impact of recurrent mitral regurgitation and ventricular remodeling on long-term outcomes in patients receiving subvalvular mitral repair combined with restrictive mitral annuloplasty versus those undergoing restrictive mitral annuloplasty alone.

## 2. Materials and Methods

### 2.1. Ethics Statement

This study is a subanalysis of a published prospective randomized clinical trial, defined as a papillary muscle approximation (PMA) trial [4], and it is included in the Transcatheter Versus Standard Surgical Mitral Valve Operation for Secondary Mitral Regurgitation (TEERMISO) registry (ClinicalTrials.gov ID: NCT05090540). All patients provided written informed consent for enrollment in the study and the surgical procedure. The study was approved by the Institutional Review Board (IRB-MTP_2022_05_202201143). The data referenced in this article will be shared upon reasonable request to the corresponding author.

### 2.2. Study Design

We investigated long-term differences in treatment failure associated with a complete surgical myocardial revascularization, including the long-term influence of residual MV regurgitation, LV remodeling, and clinical outcomes, in the 96 patients with severe chronic IMR and coronary artery disease undergoing restrictive annuloplasty alongside subvalvular repair (SV-r + RA-r group) and restrictive annuloplasty alone (RA-r group).

SMR was assessed using transthoracic echocardiography (TTE) prior to the procedure. This was based on integrative criteria adjudicated by an independent echocardiographic core laboratory. SMR was defined as an effective regurgitant orifice area (EROA) of at least 0.4 cm^2^, or by a combination of guideline-directed adjunctive echocardiographic quantification methods [10]. Coronary artery disease was evaluated using coronary angiography, and all patients achieved a complete coronary artery bypass graft (CABG) operation.

### 2.3. Study Procedure

We developed and previously published research on the papillary muscle approximation technique [3,4,11]. Briefly, standardized subvalvular repair, consisting of reapproximating both papillary muscles (PMs), was added to the standard ring restrictive annuloplasty to achieve MR correction and restoration of the subvalvular apparatus, as follows:

(1) Repositioning the PMs;
(2) Standardized realignment of the subvalvular apparatus in one plane. This is achieved by the apico-lateral realignment of both PMs to eliminate apical tenting of the anterior leaflet;
(3) Approximation of both PMs with the use of Goretex 4-0 stitch (CV-4 W.L. Gore & Associates Newark, Newark DE 19711, USA) for PMs for anatomy type 1–3 or with 4 mm Goretex graft (Gore & Associates Newark, city, DE, USA) for PMs anatomy type 4–5.

Coronary artery bypass grafting (CABG) surgery was performed using standard techniques to achieve complete revascularization with the use of single or double internal thoracic arteries (ITAs). All patients received guideline-directed medical therapy for heart failure and coronary artery disease, including antiplatelet medication, neurohormonal antagonists, lipid-lowering agents, and cardiac resynchronization and defibrillator therapy as appropriate. Patients were followed for up to 5 years after the procedure.

### 2.4. Interventions

In detail, the subvalvular repair combined with restrictive mitral annuloplasty surgery was achieved using a median sternotomy under normothermic cardiopulmonary bypass conditions and intermittent anterograde blood cardioplegia. Before starting with the cardiopulmonary bypass, transesophageal echocardiography was performed to corroborate that any mitral valve structural abnormalities occurred and all eligibility criteria were met.

End-diastolic and end-systolic interpapillary muscle distances (IPMD) were compared with preoperative values measured via transthoracic echocardiography in the parasternal short-axis view of the LV. The comparison of IPMD in physiological and pathological conditions represents a fundamental point for the success of the procedure. The interpapillary muscle distance measured in mid-systole was between 12.9 mm and 22.5 mm. It is important to highlight that these measures show a fluctuation when normalized to BSA and corrected with age. The measure of IPMD normalized to BSA and age is 10.5 mm more or 3.3 mm less (Supplementary Materials Section S1. Intervention Part).

To achieve optimal exposure of the MV through the left atriotomy, we used the Carpentier retractor (Tisurg, Jiangsu, China). At the time of the surgical inspection, all patients experienced typical lesions attributable to the Carpentier type IIIb classification that confirmed the echocardiographic diagnosis. The geometrical disorder of MV was characterized by changes to three measures: anteroposterior annular dilation, tenting area, and interpapillary muscle distance. In all patients, we noticed leaflet restriction as the result of excess traction on the leaflets, leading to a lack of coaptation.

The first step, after diastolic cardioplegic arrest, was to recognize and carefully inspect the PMs intraoperatively. IPMD was measured with a flax thread to confirm the echocardiographic findings. Variations in the measured PM distance decreased, and a second measurement was performed by the same method to corroborate the result. It is important to note that PMs which were identified anatomically as type I and II were approximated with a CV-4 Gore-Tex suture (W. L. Gore & Associates, Flagstaff, AZ, USA). The suture was arranged on the head of each PM (Supplementary Figure S1B). Considering type III, IV, or V PMs, their approximation was performed using a 4 mm Gore-Tex tube (W. L. Gore & Associates) that encircled the bodies of the posteromedial and anterolateral PMs, which were strengthened together (Supplementary Figure S1B). The identification of 2 independent heads together allowed the approximation of both posteromedial PMs to minimize MV tenting.

Particular attention was paid to the evaluation of the chordal organization to optimize the correction of regurgitation, for which the exact anatomical location of the tendon cords was evaluated. Usually, the chordae originate from the PPM and insert on the scallops P2 and P3 of the posterior leaflet; however, chordae from APM are attached to the anterior leaflet, which leads to the development of the ‘‘seagull sign’’ and respective tenting.

The second phase was the calibration of the anterior leaflet area to perform the RMA. Then, the AL was measured using a prosthetic ring obturator. Annuloplasty was undersized by 2 sizes and was performed with a Carpentier-Edwards Physio ring (Edwards Lifesciences, Irving, CA, USA). The prosthetic ring was fixed with the use of 2/0 braided sutures placed 1 mm away from the leaflet’s hinge on the atrial wall. Sutures were positioned circumferentially, starting from the posterior commissural area, in counterclockwise fashion. Larger bites were used at the posterior area of the annulus from trigone to trigone to accentuate the downsizing effect at this level. All sutures were then passed through the prosthetic ring cuff. The ring was lowered into position and the sutures were tied.

The third phase was the concomitant CABG operation, which was performed on all patients. We used a single or double internal thoracic artery (ITA) for complete revascularization of diseased coronary arteries. In patients less than 65 years of age, we preferred the use of both Y-shaped and in situ RITA and LITA.

### 2.5. Endpoints

The endpoints of this study differ from previous studies [3,4,5] and are directed to the evaluation of the impact of recurrent mitral regurgitation at up to 5 years of follow-up.

The primary endpoint was treatment failure: this was defined as a composite of death, MV reoperation, or recurrence of moderate-to-severe (3+) or severe (4+) MR within 5 years after the procedure.

Secondary endpoints included all-cause death at 30 days and within 5 years of follow-up; the echocardiographic degree of MR at discharge and 6 months, 1 year, 2 years, and 5 years; left ventricular reverse remodeling on basis of transthoracic echocardiography with particular regard to the left ventricular ejection fraction (LVEF); and the left ventricular end-systolic dimension and the functional status as assessed by the New York Heart Association (NYHA) class related to the progression of residual MR at follow-up. MR was graded according to the recommendations of the European Association of Cardiovascular Imaging using an integrative approach, which included qualitative, semiquantitative, and quantitative measures [12].

Echocardiographic endpoints, determined by by transthoracic echocardiography (TTE) examination, included the severity of the LV disease and MV tenting indicators (i.e., tenting height), which were determined at end-systole in the parasternal long-axis view. Tenting height (TH) was then measured as the distance between the mitral annular plane and the most atrial margin of the coaptation zone. The two-dimensional tenting area was fixed as the area between the mitral annular plane and the mitral leaflets at end-systole. Coaptation length was interpreted as the length of the overlap between the anterior and posterior mitral leaflet during systole. The annular plane was defined as the junction between the anterior and posterior hinge points of the mitral leaflets. Other parameters of severity were tenting angles, which were measured between the mitral leaflets, and the mitral annular plane alongside the interpapillary muscle distance, both of which were determined in the parasternal short-axis view of the left ventricle.

### 2.6. Statistical Analysis

Categorical data are displayed as frequencies and percentages in text and tables, with Fisher’s exact test or the chi-square test used as appropriate. Normality criteria were checked and met for each continuous variable. Independent sample or paired sample *t*-tests were used for normally distributed data, which are presented as mean ± standard deviation in text and tables. Incidence rate (IR) differences (IRD) were calculated for the MR and NYHA functional classes. Event-free survival curves were constructed using the Kaplan–Meier method, and treatment effects were estimated as hazard ratios with a 95% confidence interval, which was derived using the Cox proportional hazards model. The proportional hazards assumption was tested with the use of a graphical method. A multivariable adjustment was further performed with baseline age, gender, diabetes, LVEF, and severity of MR in view of the presumed association with treatment failure. Similarly, a multivariable adjustment with baseline age, gender, diabetes, and LVEF was performed to test the impact of moderate or moderate-to-severe residual MR on mortality at follow-up. A *p*-value < 0.05 was considered statistically significant. The analyses were conducted using R statistical software (version 4.1.2; R Foundation for Statistical Computing, Vienna, Austria) through the survival, survminer, and subtee packages.

## 3. Results

### 3.1. Patients

In total, 96 patients underwent randomization, 48 receiving subvalvular repair with restrictive annuloplasty repair (SV-r + RA-r group) and 48 receiving restrictive annuloplasty repair alone (RA-r group). The average native annulus size was 39.1 mm in patients who received the SV-r + RA-r and 39.5 mm in patients who received RA-r alone. The ring sizes used were 26 mm (42.7% of patients) and 28 mm (57.3% of patients). Concomitant CABG was performed in 100% of patients. There were no crossovers between the groups post-randomization. Patients underwent clinical and echocardiographic follow-ups at 6 months, 1 year, 2 years, and 5 years after the surgical procedure. The follow-ups were 100% complete. Baseline patients’ characteristics and procedural details have previously been reported [3,4] and are stratified by treatment failure in Table 1.

### 3.2. Treatment Failure at 5 Years

**Figure 1:**
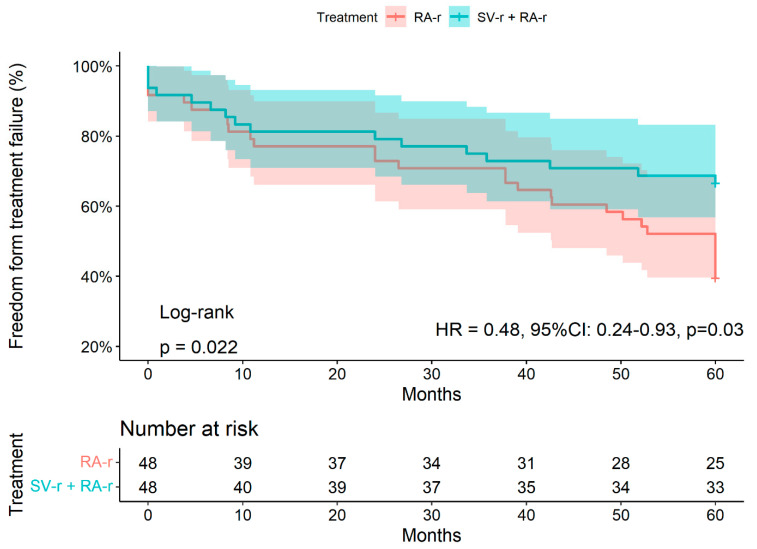
Kaplan–Meier curves for freedom from treatment failure (composite endpoint of death, MV reoperation, moderate to severe (3+) MR, or severe (4+) MR) within 5 years of follow-up after either subvalvular repair combined with restrictive annuloplasty repair (SV-r + RA-r group) or restrictive annuloplasty repair (RA-r group) alone for treatment of ischemic mitral regurgitation. Vertical marks indicate that patients’ data were censored at that point. Abbreviations: MV: mitral valve; MR: mitral regurgitation.

Within 5 years, 45/96 patients (46.9%) presented with treatment failure. Of these, 16 patients underwent SV-r + RA-r (35.6%) and 29 underwent RA-r (64,4%, *p* = 0.006, Table 1). Freedom from treatment failure at 2 years and 5 years was 66.7% (95% CI: 54.6–81.5%) and 43.7% (95% CI: 31.7–60.3%) after SV-r + RA-r versus 37.5% (95% CI: 26–54%) and 22.9% (95% CI: 13.6–38.5%) after RA-r alone, respectively (*p* = 0.02; HR adjusted 0.48, 95% CI: 0.24–0.93, *p* = 0.03, Figure 1, Supplementary Table S1).

The overall survival rates were 92% (95% CI: 88–98%) and 74% (95% CI: 66–83%) at 30 days and 5 years, respectively. Survival at 5 years was 77% (95% CI: 66–90%) in the SV-r + RA-r group and 71% (95% CI: 59–85%) in the RA-r group (*p* at log-rank test = 0.50). Within 5 years after surgery, 1 patient (7%) underwent MV reoperation in the SV-r + RA-r group, and 5 (21.7%) in the RA-r group (*p* = 0.09).

Subgroup analysis showed that the association of the treatment strategy with treatment failure was consistent across all the examined subgroups. There were no significant interactions between the impact of SV-r and ± RA-r on treatment failure within 5 years of follow-up and age (*p* = 0.36), gender (*p* = 0.6), diabetes (*p* = 0.98), baseline LVEF (*p* = 0.42), or baseline severity of MR (*p* = 0.93).

### 3.3. Influence of Residual MR

**Figure 2:**
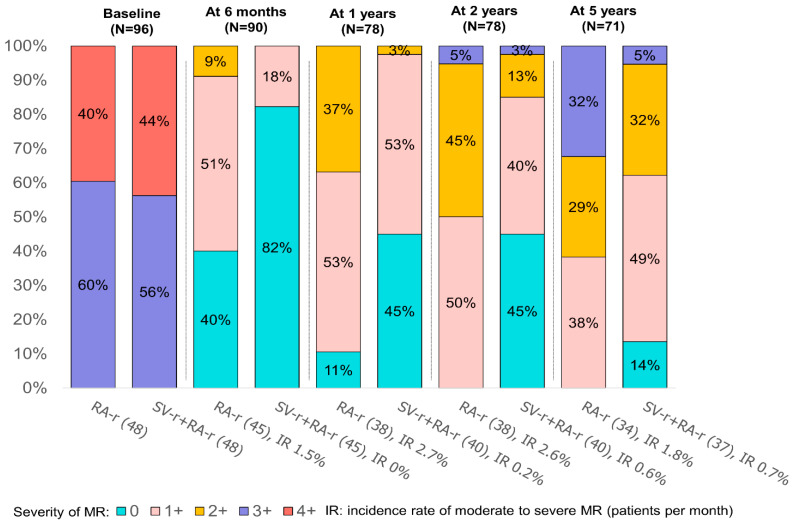
Degrees of mitral regurgitation at baseline and at 6 months, 1 year, 2 years, and 5 years after mitral valve repair, represented as percentages, in the two groups: subvalvular repair combined with restrictive annuloplasty repair (SV-r + RA-r group) and restrictive annuloplasty repair alone (RA-r group).

Overall, 94/96 patients (97.9%) had no (0) or mild (1+) residual MR at discharge. At 5 years, a moderate-to-severe (3+/4+) residual MR was observed in 5% in SV-r + RA-r versus 32% in RA-r group (*p* = 0.007, Figure 2). Interestingly, the IRs of moderate to severe MR over time were significantly lower in the SV-r + RA-r than in the RA-r group (at 24 months, IR 0.6% vs. 2.6% patients per month for SV-r + RA-r vs. RA-r, IRD *p*-value = 0.002, Figure 2).

Patients with moderate or moderate-to-severe residual MR presented a higher risk of all-cause mortality at 5 years compared with patients with no or mild MR (HR adjusted 4.67, 95% CI: 3.66–56.23, *p* < 0.001, Supplementary Table S2). Residual MR occurred earlier in the RA-r group: 20 patients in the RA-r group vs. 6 in SV-r + RA-r group had a moderate or moderate-to-severe MR 2 years after surgery (Figure 2).

### 3.4. Left Ventricular Remodeling and NYHA Class

**Figure 3:**
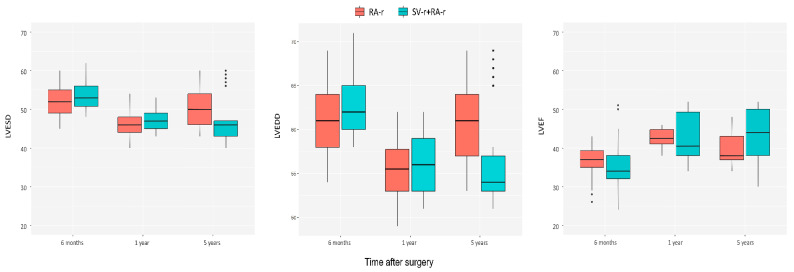
Evolution of the left ventricular end-systolic diameter (LVESD, mm), the left ventricular end-diastolic diameter (LVEDD, mm), and the left ventricular ejection fraction (LVEF, %) after mitral valve repair in the group who underwent subvalvular repair combined with restrictive annuloplasty repair (SV-r + RA-r group) and in the group who underwent restrictive annuloplasty repair (RA-r group).

Left ventricular remodeling values, recorded by TTE at 6 months, 1 year, 2 years, and 5 years of follow-up, are reported in Figure 3 and Table 2.

Left ventricular measurements, such as left ventricular diameters, tenting area, TH, interpapillary distance, and angles between the plane of the MV and the anterior and posterior leaflets, and the LVEF, worsened significantly within 5 years after surgery in patients who developed MR 2+ or 3+ for both the SV-r + RA-r and RA-r groups (Figure 3 and Table 2).

As shown in Figure 3, a reduction in the left ventricular end-systolic diameter (LVESD) and an increase in the LVEF within 5 years were observed in the SV-r + RA-r group (LVESD: 53.45 ± 3.57 mm at 6 months vs. 47.14 ± 5.91 at 5 years, *p* < 0.0001; LVEF: 365.0 ± 5.39% at 6 months vs. 44.08 ± 5.98% at 5 years, *p* < 0.0001) and in the RA-r group (LVESD: 52.22 ± 3.46 mm at 6 months vs. 50.23 ± 3.89 at 5 years, *p* = 0.02; LVEF: 36.69 ± 3.73% at 6 months vs. 39.88 ± 3.89% at 5 years, *p* = 0.0003) compared to the first echocardiographic evaluation at 6 months after the surgery.

**Figure 4:**
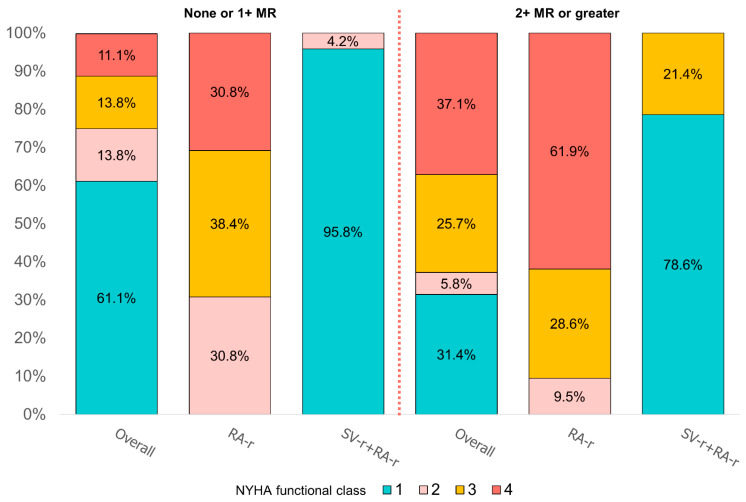
New York Heart Association (NYHA) functional class degree at 5 years according to the temporal degree of recurrence mitral regurgitation, represented as percentages, in the group of subvalvular repair combined with restrictive annuloplasty repair (SV-r + RA-r group) and in the group of restrictive annuloplasty repair alone (RA-r group), in correlation with the degree of recurrence mitral regurgitation.

At 5 years, HF symptoms, as assessed by the NYHA functional class, were available in all 71 living patients (74.0%, 37 in the SV-r + RA-r group, and 34 in the RA-r group). NYHA functional class III or IV was reported in 3 patients (8.1%) of the SV-r + RA-r group and 28 patients (82.3%) of the RA-r group (*p* < 0.0001). At 5 years, the IRs of NYHA functional classes III and IV were significantly lower (1.6% vs. 16.5% patients per year) in the SV-r + RA-r group vs. the RA-r group (IRD *p*-value < 0.001, Figure 4).

NYHA functional classes III and IV were more frequently observed in patients who had 2+ MR or greater (62.8%) than those with no or 1+ MR (25%, *p* = 0.001, Figure 4).

## 4. Discussion

Ischemic mitral regurgitation is a complex pathology associated with significant morbidity and mortality [1,2,3,4,5]. We have learned that secondary mitral regurgitation is not a valvular pathology per se, but predominantly a ventricular pathology [13,14], and that favorable ventricular remodeling and repair durability are favored by the subvalvular repair procedure [3,4,6,7,15,16,17,18]. We also learned that patients receiving surgical repair for secondary mitral regurgitation have worse outcomes at follow-up when specific valve and ventricular features are present. The risk of developing recurrent mitral regurgitation was increased with a mitral diastolic annular diameter ≥37 mm, a systolic tenting area >2.5 cm^2^, and a posterior leaflet angle >45°, indicating significant posterior leaflet restriction [5]. Additionally, severe left ventricular enlargement confers a low likelihood of reverse left ventricular remodeling after repair, as well as poor late-stage outcomes [5,19,20,21,22,23,24,25].

The current guidelines still do not include specific recommendations on the use of subvalvular repair combined with restrictive mitral annuloplasty in the treatment of ischemic mitral regurgitation. Evidence reported in the papillary muscle approximation trial, which compared subvalvular repair associated with restrictive mitral annuloplasty and restrictive mitral annuloplasty alone for patients with secondary mitral regurgitation, introduced the relative benefits of subvalvular repair combined with restrictive mitral annuloplasty for the management of severe ischemic mitral regurgitation. The trial demonstrated significant differences in reverse LV remodeling between strategies among survivors at 5 years, and SV-r combined with RA-r was associated with significantly lower rates of rehospitalization and recurrent moderate or greater MR [4]. Major differences exist between SV-r with RA-r and RA-r alone in terms of the prevalence, pathophysiology, response to surgical treatment, and prognosis across multiple long-term follow-up series. However, the use of subvalvular repair has historically been underrepresented in cardiovascular studies. Data from the literature suggest that the use of subvalvular repair plus restrictive mitral annuloplasty is less likely to be performed as surgery for the treatment of mitral regurgitation related to SMR, but may reveal better long-term outcomes than restrictive annuloplasty repair alone and mitral valve replacement [3,4,5,6,7].

In this study, the treatment failures and outcomes of subvalvular repair with and without restrictive mitral annuloplasty were compared in patients undergoing mitral valve surgery for secondary mitral regurgitation. The major findings include: (1) after adjusting for key covariates associated with each outcome of interest, restrictive annuloplasty repair remained at an increased risk of failure and mortality at 5 years compared to subvalvular repair combined with restrictive mitral annuloplasty, and the rates of recurrent mitral regurgitation were higher and occurred earlier in the RA-r group than in the SV-r + RA-r group; (2) although functional status improved in both groups after mitral valve surgery, at 5 years, the RA-r group had worse health-related and heart failure-related outcomes post-operatively than the SV-r + RA-r group; (3) LVESD, a surrogate for LV reverse remodeling, improved significantly in patients who received subvalvular repair and restrictive mitral annuloplasty compared to those who received restrictive mitral annuloplasty alone after 5 years; (4) the improvement of left ventricular remodeling is closely correlated with mitral regurgitation recurrence. The worsening of ventricular dimensions was significantly higher in patients who developed earlier 2+ or 3+ MR.

### 4.1. Treatment-Based Differences in Outcomes after Mitral Valve Surgery for Secondary Mitral Regurgitation

In the present analysis, we found that, following adjustment for baseline confounders, restrictive annuloplasty repair was associated with higher treatment failure and risk of mortality at 5 years after mitral valve surgery. The reasons for the inferior outcomes of restrictive annuloplasty repair after mitral valve surgery for secondary mitral regurgitation are clearly attributable to differences in left ventricular remodeling (measured with changes in LVESD) and to the differential grade of MR recurrence during follow-up on patients who underwent subvalvular repair plus restrictive annuloplasty compared to those who received restrictive annuloplasty repair alone. However, more importantly, in the present analysis, the absolute differences between treatment failure, recurrent MR, and death with SV-r ± RA-r appeared to be higher in the group who underwent RA-r (*p* = 0.006).

### 4.2. Influence of Recurrent Mitral Regurgitation

In our analysis, we observed that 32% of patients in the RA-r group experienced grade 3+ MR recurrence, compared to 5% in the SV-r + RA-r group. No patients had severe mitral regurgitation in the SV-r + RA-r group. These findings were seen in the RA-r group, for whom the duration of mitral repair was shortened due to recurrent mitral regurgitation. This may confer a predisposition to heart failure, atrial fibrillation, and repeat surgery or hospitalization. We noted that patients who received restrictive mitral annuloplasty had more rapid progression to 2+ or 3+ recurrent MR than patients who received associated subvalvular repair, the majority of whom developed moderate recurrent MR only in the fifth year of follow-up (*p* = 0.007).

The results reported herein are consistent with those reported in other prospective studies which demonstrated how restrictive mitral annuloplasty was insufficient to avoid recurrence of mitral regurgitation, as it led to the progressive worsening of symptoms as well as requiring reoperation after restrictive mitral annuloplasty. [5,6,8,9,21,22]. Recurrence of moderate to severe and severe MI was reported by 13.2% [3,6] and 32.6% [8] of patients at 1 year and 58.8% at 2 years [9]. These patients showed basal aneurysms, high tenting heights, preoperatively severely dilated left ventricles, and increased interpapillary muscle distances [3,4,5]. Similarly, to previous reports, this evidence translates into more severe cardiac and cerebrovascular adverse events, with a higher rate of heart failure and hospital readmission for cardiovascular causes [3,4,5,6,9].

### 4.3. Influence of LV Reverse Remodeling

In the present analysis, we observed estimated lower incidence rates of NYHA functional class III or IV (1.6% vs. 16.5% patient-years) in patients undergoing subvalvular repair plus restrictive mitral annuloplasty compared with those undergoing only restrictive mitral annuloplasty (IRD *p*-value < 0.001). Although LVESD improved significantly from the baseline in the two groups during the first year after surgery, we observed only stable improvement from baseline over the five years of follow-up in patients in the SV-r plus RA-r group. We also suggested that the worsening of ventricular dimensions was higher in the patients who developed residual MR 3+/4+, as demonstrated in the RA-r group (*p* = 0.007).

The results reported herein are consistent with those reported in our previous studies, in which restrictive annuloplasty was not able to attenuate or avoid the negative remodeling that occurred over time in SMR, and patients experienced progressive enlargement of the ventricular cavities and a decrease in left ventricular function [3,4]. Similarly, to previous reports, we found that only subvalvular repair preserved the correct LVESD and LVEDD. The restrictive mitral annuloplasty led to a high recurrence rate of MR, and it does not ensure correct ventricular dimensions over time [6,15,16,17,18].

Data from the Cardiothoracic Surgical Trials Network (CTSN) reported that mitral valve replacement was not associated with significant differences in left ventricular remodeling at 1- and 2-year follow-ups when compared with restrictive annuloplasty [8,9].

Indeed, in the CTSN trial, although the left ventricular end-systolic volume index (LVESVI) improved significantly from the baseline in the two groups during the first year after the mitral valve procedure, only a small improvement was observed during the second year [9].

Importantly, a sub-analysis of the CTSN study provided an intriguing case in point. In this trial, 74 patients with severe IMR who did not have persistent or recurrent mitral regurgitation after restrictive mitral annuloplasty had significantly smaller left ventricles at the 2-year follow-up as compared to those patients with recurrent MR after RA alone (43 ± 26 mL/m^2^ vs. 63 ± 27 mL/m^2^), and, surprisingly, compared to those patients who had undergone mitral valve replacement (61 ± 39 mL/m^2^) [9]. Therefore, the data reported by the CTSN are crucial for a better understanding of how to achieve this clinical goal of reverse LV remodeling through continued rigorous evaluation of mitral annular and left ventricular geometry relationships in patients with ischemic heart disease.

Herein, we firmly state that the possibility of achieving geometrical restoration is strictly intertwined with the regional ventricular functional status, which is responsible for PM synchrony and function. The specific regional alterations of normal LV kinesis profoundly affect the outcome of valvular and subvalvular repair. Indeed, although the dyskinesia of the inferior wall can be accommodated by the approximation of the PM, dysfunction of the anterior or lateral wall creates an excessive degree of lateral displacement and PM dyssynchrony, which cannot be compensated for by this surgical approach. Preoperative anterolateral wall dyskinesia was associated with high mortality and adverse cardiac outcomes, and we might speculate that a preoperative asymmetrical tethering pattern would be even more affected by this regional dysfunction.

The CTSN trial underwent some criticism because 26.2% of patients who received restrictive mitral annuloplasty did not have concomitant coronary surgical revascularization [9]. As it was noteworthy, in our previous report, we highlighted that for 17.9% of patients with anterior–lateral wall motion abnormality, the subvalvular repair did not confer a significant effect modifier (LVEDD −3.4 ± 7.0; *p* = 0.342) [4]. Likewise, RA-r recipients showed statistically significant adverse reverse remodeling in the presence of inferior and inferior–posterior wall motion abnormalities (*p* = 0.356 and *p* = 0.49 respectively) [4]. This evidence is in agreement with Penicka and colleagues, who revealed, in a series of moderate IMR patients receiving CABG only, that MR resolution after surgery was associated with more vital segments and less LV dyssynchrony at the baseline [26]. Not surprisingly, patients with documented scar tissue, a baseline aneurysm, dyskinesia in the lateral inferior–posterior left ventricle, large ventricles (LVESVI > 60 mL/m^2^ and LVEDD > 60 mm) [3,4,5,9], or poor coronary targets in the circumflex and right coronary distributions have a decreased probability that revascularization will provide a notable improvement in left ventricular contractility and left ventricular reverse remodeling [3,9,25,26,27,28,29].

### 4.4. Study Limitations

Besides the previously described limitations to the primary analysis [3,4], it should be noted that patients with non-ischemic dilated cardiomyopathy were not included in this analysis. Evidence has shown benefits of the use of subvalvular repair in prospective and non-randomized studies, with a limited number of retrospective controlled patients’ studies on non-ischemic dilated cardiomyopathy [17,30]. As these studies reported good results in terms of improving left ventricular reverse remodeling, additional multicenter RCTs enrolling either Carpentier class IIIb or class I patients are needed to assess the efficacy and benefit of subvalvular repair on mortality as the primary endpoint [31,32,33,34].

Finally, although the echocardiographic controls were performed by two blinded expert cardiologists, guaranteeing the reliability of the results, for the wall motion abnormalities, they were classified as inferior, infero-posterior, or anterolateral. The conventional 16-segment division used in transthoracic echocardiography would have required a much larger sample size, which was too many participants for this study to consider it as a primary endpoint.

## 5. Conclusions

SMR is a pathology that impairs the geometrical relationship between the ventricle and the MV apparatus. Reducing the size of the annulus alone does not achieve durable results over time, and results in an increased incidence of treatment failure as the composite endpoint, rehospitalization, and reintervention, primarily due to early MR recurrence. The addition of subvalvular repair improves the durability of the repair, thus extending all the benefits of preventing MR recurrence and offering a sustained improvement in echocardiographic and clinical parameters over time. Worsening MR recurrence heralds worsening LV parameters in both groups. Large-scale multicenter trials are encouraged to confirm the benefits of double-level repair as part of the armamentarium of IRM management, and to support its indication in future guidelines.

## Figures and Tables

**Table 1:** Baseline patients’ characteristics of SV-r + RA-r vs. RA-r, stratified by treatment failure.

Variables	No Treatment Failure within 5 Years (*n* = 51)		Treatment Failure within 5 Years (n = 45)		*p* Value *
	SV-r + RA-r (*n* = 32)	RA-r (*n* = 19)	SV-r + RA-r (*n* = 16)	RA-r (*n* = 29)	
Male sex	23 (71.9)	13 (68.4)	5 (31.2)	17 (58.6)	0.05
Age in years	62.88 ± 7.32	62.05 ± 5.69	62.80 ± 6.58	66.24 ± 7.98	0.15
Hypertension	18 (56.2)	5 (31.2)	10 (52.6)	13 (44.8)	0.40
Dyslipidemia	12 (37.5)	6 (37.5)	6 (37.5)	13 (44.8)	0.92
Diabetes	11 (34.3)	7 (36.8)	7 (43.8)	13 (44.8)	0.83
CKD, stage III+	4 (12.5)	2 (10.5)	5 (31.2)	9 (31.0)	0.14
Family history of CVD	8 (25.0)	3 (15.8)	4 (25.0)	9 (31.0)	0.70
COPD	3 (9.4)	1 (5.3)	4 (25.0)	5 (17.2)	0.29
Atrial fibrillation	5 (15.6)	3 (15.8)	4 (25.0)	7 (24.1)	0.76
Preoperative MR					0.97
grade 3	18 (56.2)	11 (57.9)	9 (56.2)	18 (62.1)	
grade 4	14 (43.8)	8 (42.1)	7 (43.8)	11 (37.9)	
Multivessel coronary disease	23 (71.9)	12 (63.2)	13 (81.2)	24 (82.8)	0.42
LVEF	34.78 ± 4.68	37.26 ± 2.51	35.50 ± 6.58	36.31 ± 4.34	0.28

Variables are expressed as frequency and percentage or mean and standard deviation for SV-r + RA-r and RA-r. COPD: chronic obstructive pulmonary disease; CPB: cardiopulmonary bypass; CVD: cardiovascular disease; MR: mitral regurgitation; CKD: chronic kidney disease; LVEF: left ventricular ejection fraction. * Comparison between no treatment failure vs. treatment failure; SV-r + RA-r: subvalvular repair + restrictive annuloplasty repair; RA-r: restrictive annuloplasty repair alone.

**Table 2:** LV remodeling and MR at follow-up after SV-r + RA-r and RA-r.

**Preoperative Values**	**SV-r + RA-r**			**RA-r**		
MR grade	3+/4+				3+/4+	
LVEDD	62.67 ± 3.41			61.44 ± 3.67		
LVESD	35.44 ± 3.54			52.23 ± 3.46		
LVEF	35.02 ± 5.33			36.69 ± 3.73		
Tenting area (mm^2^)	2.96 ± 0.36			2.84 ± 0.35		
Tenting height (mm)	1.21 ± 0.08			1.22 ± 0.16		
ES IPD (mm)	4.47 ± 0.42			4.42 ± 0.39		
α angle (°)	31.54 ± 2.27			31.61 ± 2.16		
β angle (°)	54.48 ± 5.00			54.10 ± 5.57		
**6 months after the operation**	**SV-r + RA-r**			**RA-r**		
MR grade	0/1+	2+/3+	*p* value	0/1+	2+/3+	*p* value
LVEDD	62.064 ± 3.41	64.14 ± 3.03	0.05	61.04 ± 3.89	61.9 ± 3.44	0.44
LVESD	52.88 ± 3.42	54.79 ± 3.58	0.09	51.4 ± 3.25	53.1 ± 3.52	0.083
LVEF	35.32 ± 5.84	34.29 ± 3.91	0.50	36.32 ± 4.33	37.1 ± 2.98	0.48
Tenting area (mm^2^)	0.83 ± 0.23	0.96 ± 0.16	0.06	0.93 ± 0.30	0.98 ± 0.21	0.55
Tenting height (mm)	0.61 ± 0.17	0.76 ± 0.18	0.01	0.68 ± 0.15	0.70 ± 0.17	0.34
ES IPD (mm)	3.13 ± 0.37	3.37 ± 0.31	0.04	4.39 ± 0.46	4.45 ± 0.30	0.57
α angle (°)	19.59 ± 4.52	21.57 ± 3.65	0.20	27.16 ± 3.61	27.6 ± 3.12	0.65
β angle (°)	38.47 ± 4.17	40.36 ± 3.48	0.10	83.32 ± 10.50	84.9 ± 9.58	0.60
**60 months after the operation**	**SV-r + RA-r**			**RA-r**		
MR grade	0/1+	2+/3+	*p* value	0/1+	2+/3+	*p* value
LVEDD	53.43 ± 1.73	61.43 ± 6.44	<0.001	58.15 ± 3.95	62.2 ± 4.32	0.01
LVESD	44.13 ± 2.22	52.07 ± 6.79	<0.001	47.62 ± 3.10	51.9 ± 4.24	0.004
LVEF	46.70 ± 4.24	39.79 ± 6.05	<0.001	41.54 ± 4.07	38.9 ± 3.48	0.001
Tenting area (mm^2^)	0.89 ± 0.13	1.28 ± 0.36	<0.001	1.9 ± 0.24	2.1 ± 0.40	0.11
Tenting height (mm)	0.64 ± 0.10	0.82 ± 0.20	<0.001	0.72 ± 0.10	0.87 ± 0.14	0.87
ES IPD (mm)	3.75 ± 0.12	4.28 ± 0.49	<0.001	4.1 ± 0.19	4.6 ± 0.61	0.007
α angle (°)	21.04 ± 2.78	23.21 ± 2.29	0.02	30.62 ± 1.26	32.6 ± 2.11	0.005
β angle (°)	37.04 ± 1.43	40.36 ± 3.48	<0.001	78.77 ± 4.66	91.2 ± 9.33	<0.001

Values are means ± SD. α: angle between the annular plane and anterior mitral leaflet; β: angle between the annular plane and posterior mitral leaflet; ES: end-systolic; IPD: interpapillary muscle distance; LVEDD: left ventricular end-diastolic diameter; LVEF: left ventricular ejection fraction; LVESD: left ventricular end-systolic diameter; MR: mitral regurgitation; SV-r + RA-r = subvalvular repair + restrictive annuloplasty repair; RA-r = restrictive annuloplasty repair alone.

## Data Availability

Nappi, Fiore, and Nenna had full access to all of the data in the study and take responsibility for the integrity of the data and the accuracy of the data analysis. The data underlying this article will be shared upon reasonable request to the corresponding author.

## Supplementary Materials

The following supporting information can be downloaded at: https://www.mdpi.com/article/10.3390/jcdd10030124/s1, Supplementary Figure S1: Double level mitral valve repair by mean of PMA + RA. The procedure reverses the mechanism of SIMR by mean of recovery of three physiological dimension: interpapillary muscle distance, tenting volume or surface and anteroposterior annular diameter; Supplementary Figure S2: Papillary muscle of mitral valve typically comprises five segmentation and morphological types; Supplementary Table S1: Multivariable Cox regression analysis for treatment failure at 5 years; Supplementary Table S2: Multivariable Cox regression analysis for mortality at 5 years. References [35,36,37,38,39,40,41] are cited in the Supplementary Materials.

## Abbreviations

CABG	coronary artery bypass grafting
CTSN	Cardiothoracic Surgical Trials Network
EROA	effective regurgitant orifice area
ES-IPD	end-systolic interpapillary muscle distance
ETT	transthoracic echocardiography
HF	heart failure
IMR	ischemic mitral regurgitation
ITAs	internal thoracic arteries
LVEDD	left ventricular end-diastolic diameter
LVEF	left ventricular ejection fraction
LVESD	left ventricular end-systolic diameter
LVESVI	left ventricular end-systolic volume index
MI	myocardial infarction
MR	mitral regurgitation
MV	mitral valve
MVR	mitral valve replacement
NYHA	New York Heart Association
PMA	papillary muscle approximation
PMs	papillary muscles
QOL	decreased quality of life
RA-r	restrictive annuloplasty repair
RCTs	randomized clinical trials
SMR	secondary mitral regurgitation
SV-r	subvalvular repair
TH	tenting height
