# Supplementary material for: Impact of Mitral Regurgitation Recurrence on Mitral Valve Repair for Secondary Ischemic Mitral Regurgitation
Source: J Cardiovasc Dev Dis. 2023 Mar 15;10(3):124. doi: 10.3390/jcdd10030124 (PMC10053850; doi:10.3390/jcdd10030124)
Supplement: Supplementary file 1 [file jcdd-10-00124-s001.zip › jcdd-2276923-supplementary.pdf]

Supplementary Materials

1. Intervention

The subannular repair (SA-r) combined with restrictive annuloplasty (RA) surgery was achieved by mean of a median sternotomy under normotermic cardiopulmonary bypass conditions and intermittent anterograde blood cardioplegia. Before starting with the cardiopulmonary bypass, transoesophageal echocardiography was performed to corroborate that any MV structural abnormalities occurred and all eligibility criteria were met.

End-diastolic and end-systolic interpapillary muscles distances were compared with preoperative values measured via transthoracic echocardiography in the parasternal short-axis view of the LV. The comparison of IPMD in physiological and pathological conditions represents a fundamental point for the success of the procedure. The interpapillary muscle distance measured in mid systole is comprised between 12.9 mm and 22.5 mm. It is important to highlight that these measures have a fluctuation when normalized to BSA and correction with age. The measure of IPMD normalized to BSA and age is 10.5 mm more or less 3.3 mm [1].

To achieve optimal exposure of the MV through a left atriotomy we used as support the Carpentier retractor (Tisurg, Jiangsu, People's Republic of China). At the surgical inspection all patients experienced typically lesions attributable to the Carpentier type IIIb classification that confirmed the echocardiographic diagnosis. The geometrical disorder of MV was chacterized by the change of three measures: anteroposterior annular dilation, tenting area and interpapillary muscle distance. In all patients we noticed leaflets restriction as the result of excess traction on the leaflets leading to a lack of coaptation.

The first step, after diastolic cardioplegic arrest, was to recognize and inspect carefully intraoperative the PMs. IPMD was measured with a flax thread to confirm the echocardiographic findings; therefore, PM distance was decreased and a second measurement was performed by the same method to corroborate the result. It is important to note PMs that were identified anatomically as type I and II were approximated with a CV-4 Gore-Tex suture (W. L. Gore & Associates, Flagstaff, Ariz). The suture was arranged on the head of each PM **(Supplementary Figure S1B).** Considering type III, IV, or V PMs, their approximation was performed using a 4-mm Gore-Tex tube (W. L. Gore & Associates) that encircle the bodies of posteromedial and anterolateral PMs, which were strengthened together **(Supplementary Figure S1B)**. The identification of 2 independent heads allowed together the approximation of both posteromedial PMs to minimize MV tenting.

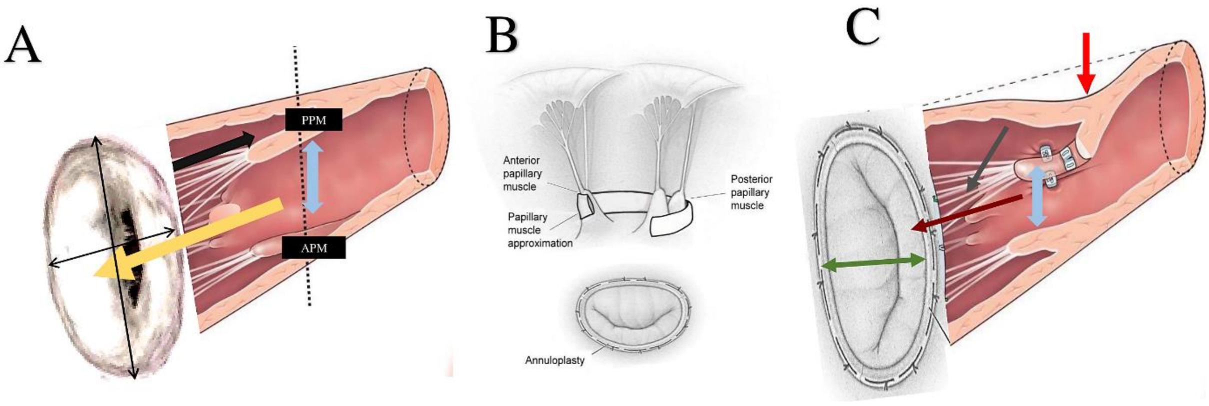

Supplementary Figure S1. Double level mitral valve repair by mean of PMA + RA. The procedure reverses the mechanism of SIMR by mean of recovery of three physiological dimension: interpapillary muscle distance, tenting volume or surface and anteroposterior annular diameter. **Figure A:** the pathoanatomical changes related to SIMR involve the mitral annulus, leaflets and subvalvular apparatus (yellow arrow). Annular dilatation (Small black arrow) is associated to leaflet tethering (Great black arrow) due to PMs displacement (blue arrow). **Figure B:** PMA was performed using a 4-mm Gore-Tex tube (W. L. Gore & Associates) that encircle the bodies of posteromedial and anterolateral PMs. **Figure C:** shows how PMA and combined RA work for the normalization of the 3 modified dimensions. 1) Corrects the displacement of PMs reducing the interpapillary muscle distance (blue arrow) that cause the leaflet tethering (Grey arrow). 2) Eliminates leaflet tenting by normalization of tenting volume or area (Purple arrow). 3) Corrects the anteroposterior annular diameter (green arrow) by mean of RA. 4) Improving of LV remodelling (Red arrow). Abbreviations: APM, anterior papillary muscle; PPM, posterior papillary muscle. Other abbreviation in teh main text.

Particular attention was paid to the evaluation of the chordal organization to optimize the correction of regurgitation, for which the exact anatomical location of the tendon cords was evaluated. Usually, from the PPM originate chordae that they insert on the scallop P2 and P3 of the posterior leaflet, whereas from APM stem the chordae destined to anterior leaflet, which lead mainly in development of the ‘’seagull sign’’ and respective tenting.

The second phase was the calibration of the anterior leaflet area to perform the RMA. Then the AL was measured using a prosthetic ring obturator. Annuloplasty was undersized by 2 sizes and was performed with a Carpentier-Edwards Physio ring (Edwards Lifesciences, Irving, Calif). The prosthetic ring was fixed with the use of 2/0 braided sutures placed 1 mm away from the leaflet’s hinge on the atrial wall. Sutures were positioned circumferentially starting from the posterior commissural area in counterclockwise fashion. Larger bites were used at the posterior part of the annulus from trigone to trigone to accentuate the downsizing effect at this level. All sutures were then passed through the prosthetic ring cuff. The ring was lowered into position and the sutures were tied.

The third phase was the concomitant CABG operation which was performed in all patients. We used a single or double ITA for complete revascularization of diseased coronary arteries. In patients less than 65 years of age we preferred the use of both Y-shaped and in situ RITA and LITA

2. Technical consideration of intervention.

2.1. Papillary Muscle Anatomy and Vascularization

We recognize five segmentation and morphological types of papillary muscle of mitral valve. The most usual patterns include: type I, single uniform unit; type II, groove with two apexes; type III, fenestrations with muscular bridges ; type IV, complete separation in two adjacent heads ; and type V, complete separation with two distant heads **(Supplementary Figure S2A)**. An appropriate analysis during the papillary muscle handling needs knowledge about its division that occur according two directions corresponding to a sagittal and coronal plane and or to coronal plane **(Supplementary Figure S2B)** [2–5].

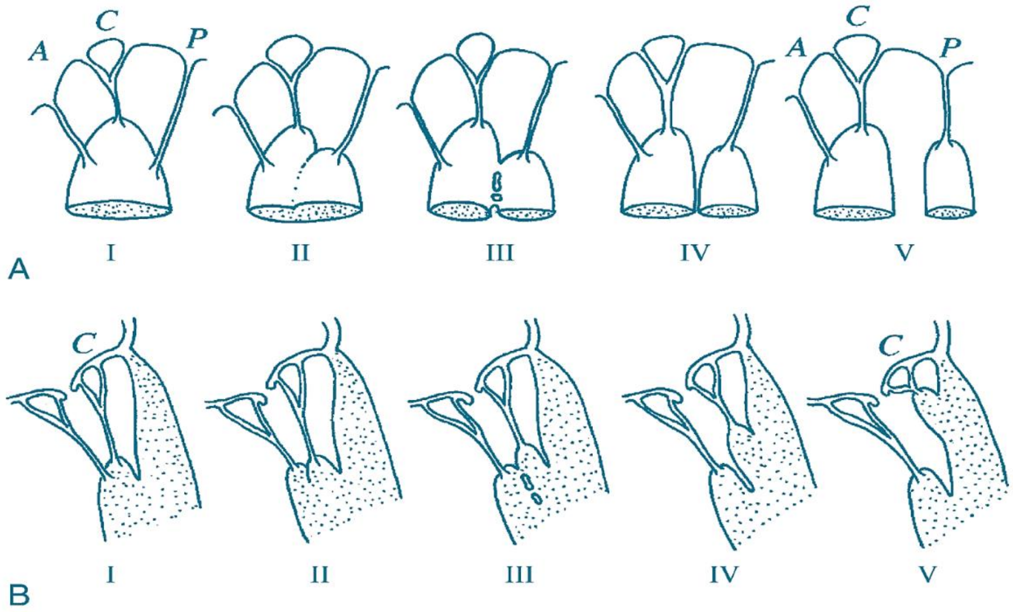

Supplementary Figure S2. Papillary muscle of mitral valve typically comprises five segmentation and morphological types. The most common patterns are reported in **Figure A**: type I, single uniform unit; type II, groove with two apexes; type III, fenestrations with muscular bridges; type IV, complete separation in two adjacent heads; and type V, complete separation with two distant heads. An appropriate analysis of the papillary damage must include the division that can occur according to two directions corresponding to a sagittal plane as showed in **Figure A** or to a coronal plane as showed in **Figure B**. A, anterior leaflet; C, commissure; P, posterior leaflet.

With regard to the distribution of coronary blood flow in the papillary muscles we descibed a prevalence of scarce vascularization of PPM among persons with a ischemic mitral prolapse ranging about 91%. This variation is due to the different coronary vascular distribution in the papillary muscle. The existence of an uneven distribution of the blood supply explains the rare involvement of the anterior PM and of the whole segment of the ventricular wall. In fact, the perfusion of this muscle is guaranteed both by the left anterior descending coronary artery and by the diagonal branch. In addition, from biomechanical point of view the evidence suggest for a tension exerted by the chordae on APM leading to a relatively low dynamic stress-strain due to its superficial location with regard to the annulus.

On the contrary, the difference experienced in the PPM injury is related to greater sensitivity to ischemia (reported 91% of the cases in our works) because this muscle is perfused by either the right coronary or the circumflex artery. Moreover, from biomechanical point of view the higher stress recorded in PPM is strongly associated to its location deep in the left ventricle leading to higher shear force.

Analysis of PM microcirculation evokes an independent blood supply assured by a segmental distribution and a well-identified arterial trunk, named Kugel's artery that perforates the PM from base to apex. This finding relative to microcirculation and anatomical characteristics of PM allocate the relative importance of one of the two circulatory systems. Frailty of PM depends on the morphology and position of the PM within the ventricle and on the presence of muscular bridging, which favors collateralization. The evidence supporting the importance of the truncal system increases when the PM is more individualized from the ventricular wall, as in type IV–V. In this pattern the apex becomes more prone to rupture due to the fragility of its truncal blood supply and to the degree of physical stress.

2.2. Surgical consideration related to the technique

The intraoperative goal was to achieve reducing IPMD by an average value of 30% of the end-diastolic interpapillary distance compared to preoperative value (flax thread method), confirmed via the use of intraoperative transoesophageal echocardiography. The degree of reduction achieved led to local remodeling of the LV. A further rapprochement of the papillary muscles can be determined by the overall favorable recovery of left ventricular remodeling. This occurs because the beneficial effects attributable to reverse remodeling of the left ventricle led to a reduction in IPMD.

Some factors must be considered during the procedure of rapprochement. The first point is the quantification of MR that is achieved in endsystole with a variation of IPMD comprise between 17 mm and 22 mm. The second point concerns the treatment of the papillary muscle which is performed during the operative ventricular diastole, during which IPMD is closely related to the dilation of the left ventricular chamber. IPMD measured in enddiastole is greater than in endsystole when measurements are obtained to quantize mitral regurgitation. Finally, the reduction of the LV size leads to an additional IPMD approach.

Authors considered the option of PM relocation in patients who had SIMR [6,7], however, we have ruled out taking this path because we believe that this technique does not address the multidirectional displacement and migration of the PM and might result in increased tension at the level of the posterior trigone and potential adverse biomechanical effects. Indeed, Watanabe found that the of the relocation of the PPM might be associated with a restrictive effect on the MV if directed only to the posterior leaflet as inducing a tilting effect on the posterior annulus with increased posterior tethering. In these cases, assume the great importance the anatomy of PPM especially in its V form which identify two distinct heads for the PM and relative chordae for both leaflets. Therefore, the relocation of both PMs that is absolutely required [6].

Supplementary Table S1. Multivariable Cox regression analysis for treatment failure at 5 years.

**Characteristic**		**HR[1]**	**95% CI[1]**	**p-value**
**Procedure**	RA-r		ref	
	SV-r+RA-r	0.48	0.24, 0.93	0.030
Age		1.02	0.97, 1.07	0.4
Male sex		0.54	0.28, 1.05	0.071
Diabetes		1.00	0.53, 1.87	>0.9
Preoperative Left Ventricular Ejection Fraction		0.98	0.89, 1.06	0.6
Preoperative MR grade	3+		ref	
	4+	1.38	0.70, 2.69	0.4
HR[1] = Hazard Ratio, CI[1] = Confidence Interval, MR=Mitral Regurgitation, ref means the reference for MR grade 4+ vs 3+.				

Supplementary Table S2. Multivariable Cox regression analysis for mortality at 5 years.

**Characteristic**		**HR[1]**	**95% CI[1]**	**p-value**
**Procedure**	RA-r	ref		
	SV-r+RA-r	0.32	0.12, 0.84	0.021
Age		1.06	0.99, 1.14	0.081
Male sex		0.48	0.19, 1.19	0.11
Diabetes		0.97	0.42, 2.22	>0.9
Preoperative Left Ventricular Ejection Fraction		0.90	0.80, 1.00	0.047
Moderate or moderate to severe residual MR		4.67	3.66, 56.23	<0.001
HR[1] = Hazard Ratio, CI[1] = Confidence Interval, MR= Mitral Regurgitation				

Supplementary References

1. Sonne, C.; Sugeng, L.; Watanabe, N.; Weinert, L.; Saito, K.; Tsukiji, M.; Yoshida, K.; Takeuchi, M.; Mor-Avi, V.; Lang, R.M. Age and body surface area dependency of mitral valve and papillary apparatus parameters: assessment by real-time three-dimensional echocardiography. *Eur. J. Echocardiogr*. **2009**, *10*, 287–294.
2. Nappi, F.; Nenna, A.; Spadaccio, C.; Lusini, M.; Chello, M.; Fraldi, M. Predictive factors of long-term results following valve repair in ischemic mitral valve prolapse. *Int. J. Cardiol.* **2016**, *204*, 218–228.
3. Nappi, F.; Cristiano, S.; Nenna, A.; Chello, M. Ischemic mitral valve prolapse. *J. Thorac. Dis.* **2016**, 8, 3752–3761.
4. Nappi, F.; Spadaccio, C. Obstructive Cardiomyopathy and Tethering in Ischemic Mitral Regurgitation: Two Sides of the Coin. *Ann. Thorac. Surg.* **2019**, 107, 1911–1912
5. Jouan, J.; Tapia, M.; CCook, R.; Lansac, E.; Acar, C. Ischemic mitral valve prolapse: mechanisms and implications for valve repair. *Eur. J. Cardiothorac. Surg*. **2004**, 26, 1112–1117.
6. Watanabe, T.; Arai, H.; Nagaoka, E.; Oi, K.; Hachimaru, T.; Kuroki, H.; et al. Influence of procedural differences on mitral valve configuration after surgical repair for functional mitral regurgitation : in which direction should the papillary muscle be relocated ? *J. Cardiothorac. Surg.* **2014**, *9*, 185.
7. Fattouch, K.; Castrovinci, S.; Murana, G.; Dioguardi, P.; Guccione, F.; Nasso, G.; et al. Papillary muscle relocation and mitral annuloplasty in ischemic mitral valve regurgitation: midterm results. *J. Thorac. Cardiovasc. Surg.* **2014**, *148*, 1947–1503.
